# Barriers and facilitators for colorectal cancer screening in a low-income urban community in Mexico City

**DOI:** 10.1186/s43058-020-00055-z

**Published:** 2020-07-10

**Authors:** Karla Unger-Saldaña, Minerva Saldaña-Tellez, Michael B. Potter, Katherine Van Loon, Betania Allen-Leigh, Martin Lajous

**Affiliations:** 1 National Council of Science and Technology – National Cancer Institute, Mexico City, Mexico; 2 Epidemiology Research Unit, National Cancer Institute, Mexico City, Mexico; 3 Department of Family and Community Medicine, UCSF School of Medicine, San Francisco, USA; 4 UCSF Helen Diller Family Comprehensive Cancer Center, San Francisco, USA; 5 Center for Research on Population Health, National Institute of Public Health, Mexico City, Mexico; 6 Department of Global Health and Population, Harvard T.H. Chan School of Public Health, Boston, USA

**Keywords:** Colorectal cancer, Cancer screening, FIT test, LMICs, Barriers implementation science, Qualitative methods, Social ecological model, PRECEDE-PROCEED

## Abstract

**Background:**

Colorectal cancer (CRC) incidence and mortality are increasing in many low- and middle-income countries (LMICs), possibly due to a combination of changing lifestyles and improved healthcare infrastructure to facilitate diagnosis. Unfortunately, a large proportion of CRC cases in these countries remain undiagnosed or are diagnosed at advanced stages, resulting in poor outcomes. Decreasing mortality trends in HICs are likely due to evidence-based screening and treatment approaches that are not widely available in LMICs. Formative research to identify emerging opportunities to implement appropriate screening and treatment programs in LMICs is, therefore, of growing importance. We sought to identify potential barriers and facilitators for future implementation of fecal immunochemical test (FIT)-based CRC screening in a public healthcare system in a middle-income country with increasing CRC incidence and mortality.

**Methods:**

We performed a qualitative study with semi-structured individual and focus group interviews with different CRC screening stakeholders, including 30 lay people at average risk for CRC, 13 health care personnel from a local public clinic, and 7 endoscopy personnel from a cancer referral hospital. All interviews were transcribed verbatim for analysis. Data were analyzed using the constant comparison method, under the theoretical perspectives of the social ecological model (SEM), the PRECEDE-PROCEED model, and the health belief model.

**Results:**

We identified barriers and facilitators for implementation of a FIT-based CRC screening program at several levels of the SEM. The main barriers in each of the SEM levels were as follows: (1) at the social context level: poverty, health literacy and lay beliefs related to gender, cancer, allopathic medicine, and religion; (2) at the health services organization level: a lack of CRC knowledge among health care personnel and the community perception of poor quality of health care; and (3) at the individual level: a lack of CRC awareness and therefore lack of risk perception, together with fear of participating in screening activities and finding out about a serious disease. The main facilitators perceived by the participants were CRC screening information and the free provision of screening tests.

**Conclusions:**

This study’s findings suggest that multi-level CRC screening programs in middle-income countries such as Mexico should incorporate complementary strategies to address barriers and facilitators, such as (1) provision of free screening tests, (2) education of primary healthcare personnel, and (3) promotion of non-fear-based CRC screening messages to the target population, tailored to address common lay beliefs.

Contributions to the literature

- In low- and middle-income countries, there is a gap in the integration of qualitative research findings into the design of sustainable cancer screening programs.
- Formative research to identify context-specific barriers and facilitators to CRC screening in middle-income countries will be critically important to guide the successful design and implementation of new culturally appropriate screening programs.
- Our study highlights the relevance of qualitative methods to uncover context-specific barriers and facilitators as perceived by the different stakeholders as a critical step in the design of health interventions in a middle-income country.

## Background

Colorectal cancer (CRC) is preventable and curable with screening and early detection, yet it remains a leading cause of cancer mortality worldwide [1]. CRC incidence and mortality varies by country and region but is increasing in low- and middle-income countries (LMICs) while decreasing trends have been observed in some high-income countries (HICs) [2]. CRC results in approximately 880,000 deaths per year globally, with most of these deaths occurring in LMICs [3]. CRC screening reduces incidence and mortality by allowing detection of pre-malignant lesions that can be removed before they become cancerous, as well as by detection and removal of early-stage cancers that are curable [2]. The available evidence suggests that most deaths from CRC could be avoided with implementation of screening programs, as indicated by the decreasing trends in CRC incidence and mortality in some HICs [2, 4].

Evidence from randomized clinical trials has formed the basis for international guidelines recommending CRC screening for at-risk adults utilizing different approaches to screening, including stool-based tests and visual exams of the colon and rectum [4–7]. Stool-based tests, including the fecal immunochemical test (FIT), guaiac-based fecal occult blood test (gFOBT), and fecal DNA tests, are highly sensitive and non-invasive. However, they lack specificity, require serial testing at short intervals, and a subset of patients must still undergo direct visualization following receipt of a positive result. Visual exams of the colon and rectum, including colonoscopy, flexible sigmoidoscopy, and computed tomographic colonography offer enhanced specificity but are more invasive and costly. In resource-constrained settings, the use of non-invasive stool tests offers the advantage of higher screening uptake and lower demand on endoscopy resources [4].

CRC screening recommendations and screening programs are highly variable around the world, in part due to variations in CRC incidence, economic resources, and healthcare infrastructure [4]. In general, organized population-level CRC screening programs only exist in HICs, mainly in Western Europe, Japan, Australia, and several provinces of Canada [4]. Other HICs, most notably the USA, have achieved high levels of screening through decentralized screening programs organized at the level of public and private health systems. The reasons for the recent declining trends in incidence and mortality in some of these countries are ill-defined but are thought to be a consequence of increased early detection and removal of precancerous polyps, as well as early detection of early-stage cancers. Efforts to establish organized CRC screening programs are emerging in Latin American countries with increasing incidence of CRC, including Argentina, Brazil, Chile, and Uruguay [4]. In Mexico, CRC incidence and mortality are on the rise, possibly due to the combined effect of changing lifestyles and the improvement of healthcare infrastructure to facilitate diagnosis [8]. Unfortunately, a large proportion of cases are diagnosed at advanced stages [9], and CRC is the leading cause of cancer-related death in Mexico City [10]. Even though the Mexico’s National Clinical Practice Guidelines recommend annual gFOBT for average-risk individuals, efforts to formally implement CRC screening programs in Mexico are nascent [11].

Barriers and facilitators to CRC screening in middle-income countries (MICs) may be quite different from HICs, due to differences in health care infrastructure, resources, population characteristics, or other factors. Formative research to identify context-specific barriers and facilitators to CRC screening in MICs will therefore be critically important to guide the successful design and implementation of new screening programs in these settings. Mexico City has both a higher known incidence of CRC and more clinical resources for diagnostic confirmation and treatment compared to other regions of Mexico. Therefore, as a first step to identify promising opportunities to develop effective CRC screening programs for Mexico, we chose to begin our investigation in Mexico City.

## Methods

### Study setting and design

We undertook a qualitative study according to the standards for reporting qualitative research (SRQR) (this guide is available as supplementary material) [12]. We collected data using semi-structured individual and focus group interviews with lay people at average risk for CRC, healthcare personnel from a local public clinic, and personnel from an endoscopy unit in a cancer referral hospital.

We selected a low-income urban community of approximately 20,000 people located in the Tlalpan district of Mexico City. We chose this community because of its high levels of marginalization, capabilities of the community-based clinic, and proximity and accessibility to the Instituto Nacional de Cancerlogía (INCan), a national cancer referral hospital with an Endoscopy Unit in Mexico City (approximately 10 kilometers and 40 minutes away via public transportation). The community clinic selected (*Cultura Maya* Clinic) provides services for uninsured patients and those covered at the time by a governmental health insurance program for people without social security called *Seguro Popular*. The clinic employed 11 physicians, 16 nurses, and 7 social workers and offers free primary care services, basic x-ray imaging, and routine laboratory tests; CRC screening is not currently offered as a part of routine care. At the time of this study, the clinic served an estimated 4,213 adults between the ages of 50 to 74, which is considered as the population at-risk for CRC according to US guidelines [13].

### Study participants

We had three groups of participants. The “community participants” group was composed of lay people residing close to Cultura Maya health center. The “primary healthcare participants” included healthcare personnel employed at the Cultura Maya clinic (i.e., social workers, nurses, and primary care physicians). Finally, the “endoscopy unit participants” were healthcare personnel employed at INCan’s Endoscopy Unit (i.e., endoscopists, nurses and screening program administrative personnel). Tables 1 and 2 summarize participants’ characteristics.

**Table 1:** Characteristics of community participants (*n* = 30)

	Num.	%
**Age*(mean, range)***	*64.3 (49–80)*	
**Sex**		
Female	22	70.0
Male	9	54.9
**Marital status**		
In a cohabiting relationship	20	66.7
Not in a cohabiting relationship	10	33.3
**Illiterate**		
Yes	21	70.0
No	9	30.0
**Education**		
None	5	16.6
6 years or less	17	56.7
7 to 9 years	6	20.0
10 years or more	2	6.7
**Monthly family income**		
< 1 minimum wage salary*	18	60.1
2–3 minimum wage salaries	8	26.6
> 3 minimum wage salaries	1	3.3
No response	3	10.0

*One minimum wage salary in 2018 in Mexico City was equivalent to $139.2 USD per month

**Table 2:** Characteristics of healthcare personnel who participated in interviews (*n* = 20)

	Num.
**Sex**	
Female	13
Male	7
**Health care facility**	
Primary care clinic	13
Endoscopy Unit	7
**Job**	
Primary care physician	4
Primary care nurse	4
Primary care social worker	5
Endoscopist	5
Chief nurse at endoscopy unit	1
Coordinator at endoscopy unit	1

### Theoretical perspectives

Our study was guided by the broad theoretical perspectives of the social ecological model (SEM), the Predisposing, Reinforcing and Enabling Constructs in Educational Diagnosis and Evaluation - Policy, Regulatory, and Organizational Constructs in Educational and Environmental Development model (PRECEDE-PROCEED), and the health belief model (HBM). While there are many theories, models, and frameworks that have been used in implementation science studies, there is no consensus in the criteria for selecting the best one [14]. For this study, we selected our theoretical approaches for optimization of generalizability, process guidance, and application to our specific setting [15].

We chose SEM because it emphasizes the interaction and interdependence between factors within and across all levels of a health behavior, in this case CRC screening: intrapersonal or individual, interpersonal, institutional or organizational, community, and public policy levels. The main postulate of SEM is that behaviors both shape and are shaped by the social environment [16, 17]. We decided to strengthen the analysis of individual level factors using the health belief model (HBM), which has been widely used in the field of social psychology to explain and predict health-related behaviors. This model stipulates that people’s beliefs about a health problem (perceived susceptibility and perceived severity), the perceived benefits of and barriers to action, and self-efficacy, explain adoption (or lack of adoption) of health-promoting behaviors [18, 19]. The HBM has been mainly used to understand barriers and facilitators of cancer screening participation and to design implementation programs that enhance participation [20]. Finally, PRECEDE-PROCEED has been widely used for planning health programs. This model explains behavioral change as the result of the interplay of predisposing factors that motivate the behavior, enabling factors for the actual realization of the health behavior, and factors that reinforce the individuals’ decision to adopt and maintain the desired behavior [21, 22].

We used the three models to develop our interview guides and to inform the analysis. Additionally, we used PRECEDE-PROCEED and SEM to organize our results focusing on identifying barriers (predisposing factors) and facilitators (enabling factors), at the different levels of the SEM.

### Data collection

We used semi-structured interview guides with open-ended questions to ask participants about their perceptions of barriers and facilitators, knowledge, attitudes, and beliefs about CRC and CRC screening and strategies for motivating behavior change among lay people and health personnel. We prepared our interview guides based on the selected theoretical frameworks and key findings from the existing literature on barriers and facilitators for CRC screening [23–30].

Data were collected between September 2018 and January 2019. We conducted a total of 22 semi-structured interviews and three focus group interviews with 28 community participants, to achieve saturation with a total of 50 participants: 13/22 interviews were with primary care personnel, 7/22 with endoscopy unit participants, and 2/22 with lay members of the community.

Community participants were purposefully sampled to include a balanced perspective of men and women, who lived close in the clinic catchment area and were in the at risk age group. Participants were recruited in several ways: (1) during field visits to the local clinic, one of the researchers spoke about the study to people that seemed to be within the target age range (between 50 and 75) and took their contact data to invite them to a focus groups later on; (2) candidates were also invited by social workers from *Cultura Maya* and two other smaller health clinics nearby; and (3) key community members with local leadership who were introduced to the researchers by social workers of the primary care clinics also invited potential candidates. All healthcare participants were invited personally by one of the researchers and the interview was scheduled according to their availability.

We stratified community focus groups by gender in an effort to facilitate a more open discussion. We conducted two focus groups with women (20 total participants) and one with men (8 participants). The focus groups were led by two experts in qualitative research (KUS and MST), with one moderating and the other assisting with organization and on-site coding of responses. Written informed consent was obtained from all participants prior to the interviews. Written information was given to all of them and read out loud by one of the researchers in the presence of a witness (usually another participant or a participant’s relative). All participants provided written informed consent following an opportunity to ask questions regarding the study. For participants with low literacy who were unable to sign, a stamped fingerprint was used in lieu of the signature. We also collected demographic data from all community participants including age, marital status, years of education, family income, and household characteristics.

After the initial open questions regarding knowledge about CRC and CRC screening, we provided basic information on these topics to the participants in order to elicit their perceived barriers and facilitators for implementation of FIT-based screening and colonoscopy. The interviews with primary care providers and community participants took place in a private room at the community health clinic in Tlalpan and those with endoscopy personnel at the INCan Endoscopy Unit. Individual interviews lasted between 30 and 60 min, and focus groups lasted between 60 and 90 min. Community participants received a gift card valued at 10 USD as a small token of compensation. All interviews were carried out in Spanish, audio recorded, and transcribed verbatim.

### Data analysis

All transcripts were de-identified prior to analysis. Transcripts and field notes were organized with Atlas.ti software to aid the analysis. Data was coded by two researchers (KUS, MST) using the constant comparative analysis method, without following all classic Grounded Theory procedures, as have other authors [31]. We used this approach instead of approaching data analysis without having reviewed the literature, as classically proposed in Grounded Theory by Glaser, instead following Strauss and Corbin’s suggestion to do a review of the literature before the analysis in order to enhance theoretical sensitivity [32–34]. The constant comparative analysis method is an iterative and inductive process of reducing the data through constant recoding [32]. Data are compared to other data during the process of coding within a single interview, between interviews within the same group and between interviews from different groups. Constant comparison assures that all data are systematically compared to all other data in the data set [35]. We used a pragmatic approach for data interpretation [36, 37], identifying barriers, facilitators, and possible implementation strategies, under the theoretical lenses of SEM, PRECEDE-PROCEED, and HBM when coding and comparing our data. Data saturation was achieved with the last focus group and, therefore, no more participants were recruited. To determine saturation, we used the on-site coding to determine when no new codes appeared and each of the codes had been applied to a sufficient amount of data.

To enhance trustworthiness and rigor, we used triangulation for coding of the data. Data were coded by two different researchers from unique backgrounds. One (MST) is a social psychologist with postgraduate studies in health psychology and the other (KUS) is a medical doctor and health systems researcher. The coding results were then reviewed with adjudication in the cases of differing results, reaching consensus between the two coders to establish the final codes. Although we are both female, we felt the male informants spoke very openly to us in regard to their perceptions, as can be seen in several of the selected participant quotes. Neither of the two researchers who coded the data had previous links with the clinic or the community, and therefore, it is unlikely that participants’ responses were influenced by our presence.

## Results

**Fig. 1:**
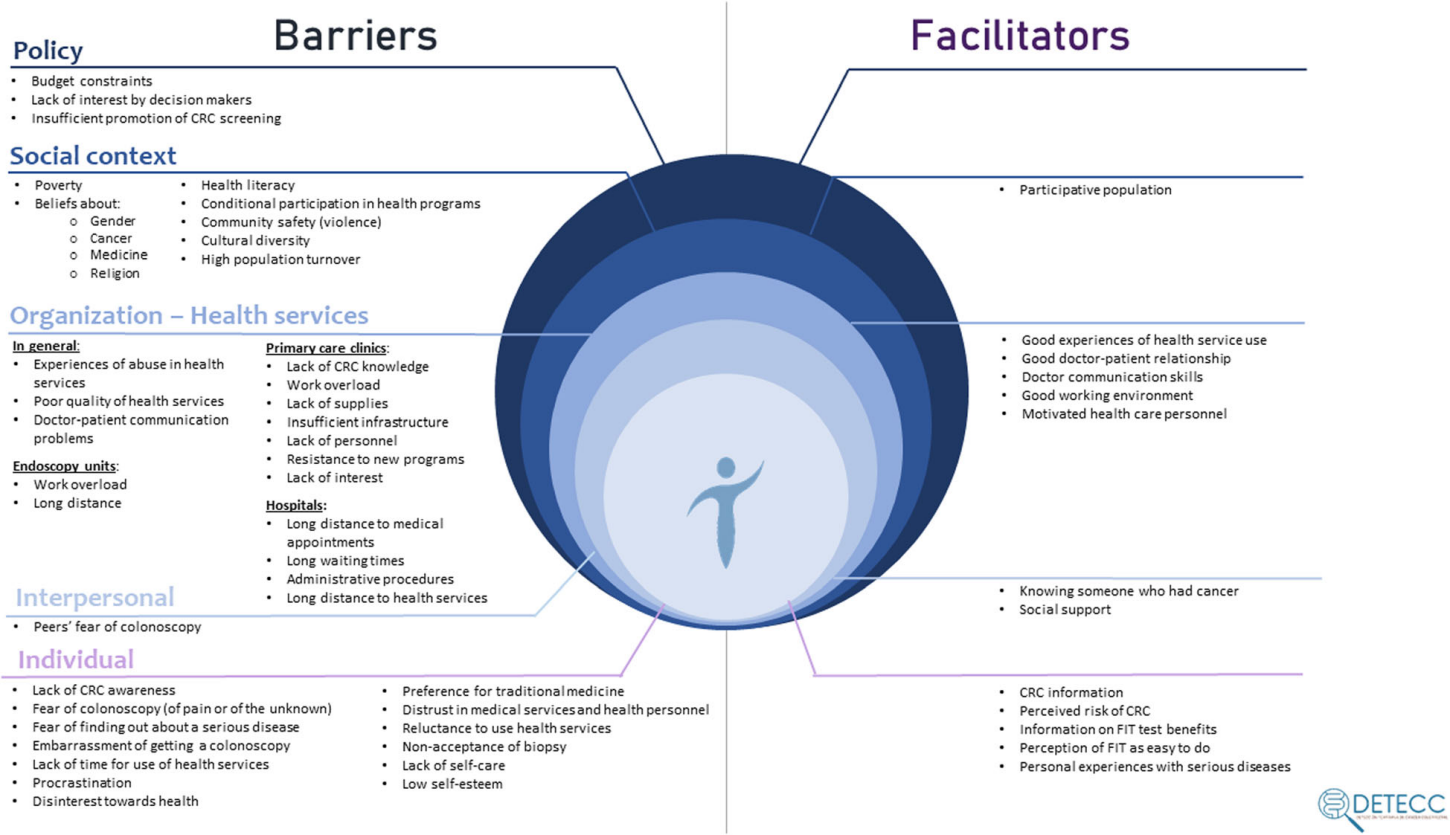
Findings of the perceived barriers and facilitators for participation in a CRC screening program in this community at the different levels of the SEM, where all levels interact with each other

A total of 30 community members and 20 healthcare providers participated in the study. Participant characteristics are summarized in Tables 1 and 2. Following the PRECEDE-PROCEED model, we organized our findings into two broad categories: (1) barriers and (2) facilitators of CRC screening, and an additional subsection that includes implementation strategies that were suggested by the participants. Additionally, each barrier and facilitator was classified within a level of the SEM. Figure 1 summarizes our findings of the perceived barriers and facilitators for participation in a CRC screening program in this community at the different levels of the SEM, where all levels interact with each other. Representative examples of participants’ quotes for the most relevant codes are presented in Tables 3 and 4.

**Table 3:** Participant quotes of perceived barriers to colorectal cancer screening

Code	Informant	Quote
***Health policy level***		
Budget constraints	Endoscopy medical personnel	We are historically used to investing in resources for treatment of advanced colon cancer, palliative care, chemotherapy, radiotherapy, studies for staging and follow-up, but we have not invested in prevention and diagnostics. We need to guarantee the funding of programs like this, and find out how much they can actually expand otherwise the effort will be useless. Its funding needs to be guaranteed by *Seguro Popular* or something of the sort for the (screening) program to keep running.
Lack of interest of decision makers	Endoscopy medical personnel	This kind of program could fail due to the lack of support of decision makers. I see that many authorities are not interested in colorectal cancer, they don’t think for a minute about the possibility of having a prevention program. Some authorities in our hospital are aware of the relevance of this, but not all...
Insufficient promotion of CRC screening	Endoscopy medical personnel	The main challenge is that more people need to get into the program. We need a lot of promotion, a lot of promotion, a lot of promotion, we need to increase the number of participants, yes!
***Social context level***		
Poverty	Male lay participant	I owed the payment of two months of my water service. So I saved money, about 1000 pesos (USD 50), but still I could not pay it. Somebody told me I could get a discount with my elderly person card, but it turned out that it only works for transportation. I had to pay 2026 pesos... I told him I have nothing more, so I just paid what I could and I still owe 1026 pesos.
	Primary care personnel: doctor	The people are humble, they are very noble people... but when you talk to them about their children’s nutrition and insist that they need to feed their malnourished children, they cannot stand it, they ask you: what can I feed them if I only have an egg for each day?
Beliefs: gender	Male lay participant	Men my age and older, it is very difficult that they will agree (to having a colonoscopy), because they are going to say that they are being raped. They will say: at this age they are going to rape me with the finger? No, you are crazy, I tell you the truth.
	Female lay participant	We are far from many things, because first we start under the assumption that us women are destined to be nothing more than a housewife, and if you have a controlling and jealous husband, forget it, how do you think you are going to go get this test done?
Beliefs: cancer	Female lay participant	I was told by a doctor that sometimes women can get colorectal cancer because when you are in labor you push a lot, and pushing so hard brings problems in the colon.
Health literacy	Primary care personnel: doctor	The patients have low levels of school education, people with maximum 3 years of primary school, so we face many complications because they do not understand how to take the treatment or how to take samples for lab tests.
Conditional participation in health programs	Primary care personnel: doctor	Above all, I have seen it happen a lot in the pap smear tests. It is very common that the results are not picked up, despite the fact that they are visited at their homes, that they receive phone calls... it is a lack of interest because they have already received the money and everything they can get and that’s it.
Insecurity (violence)	Primary health care: social worker	I feel, well not feel, I have perceived that many people, maybe approximately sixty percent, has had or has a relative in jail, and I don’t think that it’s without reason. In the area there has been violence... any act or infraction committed by their relatives or themselves is because there has been violence.
Cultural diversity	Primary health care: social worker	In this particular area there are several different cultures, I see it within the same group of people. One lady behaves differently from the other and suddenly there are many cultural shocks, right? Because there is one that says: “this is how things are done,” and there is another one that says: “no, how can you think that?”... I think this makes it difficult to form community groups in this health center... this is a particular situation that I see in this area.
Floating population	Primary health care: social worker	While the patients are from this health center, as long as they do not change residence there is no problem for the follow-up ... but it is common that they move, change their address. So you go looking for them and they are gone and you don’t know where they moved to... we have to deliver results and they are no longer there.
***Organizational level (health services)***		
Tests costs	Male lay participant	If the cost of the tests is covered by *Seguro Popular* or if the cost is low, it is most likely to be done, if it is not covered or if the cost is very high, it is difficult for a patient to perform it.
Experiences of abuse in health services	Male lay participant	It is true that security guards are sometimes very bad, completely inhuman, right? They say: you are not from around here, you need to show me your health service identity card, if not then look elsewhere... If it is already a hardship to get to one hospital, then imagine having to move from one place to another.
Experiences of abuse in health services	Primary health care: social worker	If they would explain to the patient what they are going to do to him, the patient would be relaxed, he would know that there is not going to be a bigger problem, right? But no, they go “let’s see, get on here, take your clothes off, hold on and shut up!”
Poor quality of health services	Primary care personnel: doctor	As health personnel, we have to keep up-to-date in our medical knowledge, but when they send us to training for this, it further reduces our time for patient care
Doctor-patient communication problems	Male lay participant	I did not understand. I got confused. Apparently the doctor sent me to pathology, and he sent me to do some chest studies, and some study of my entire skeleton. But I got a bunch of papers and did not understand what they were for. I thought that I had to bring back those same papers when I came back to the hospital in my next visit. It was not like that... they were for the studies. Now I pay more attention to what this is and that... They give you so many different papers.
Lack of CRC knowledge	Primary care personnel: doctor	I think we need more information, we need more in the sense that maybe, well in my case, I do not know anything about the disease and about the test, I do not know about which one is the best. I think the specialist that has to do the study would be the proctologist, but I do not have more information, nothing more. I would say that in my service we require more information.
Work overload	Primary care personnel: doctor	We’re only a nurse, a doctor, a social worker and a lot of people, so obviously you cannot cope with the care required by all the patients who come. You have to organize your times, because there are so many activities to cover. You know that if a procedure gets a bit complicated or takes you a little extra time, you will not be able to perform two or three pap smears. I would like to be able to organize my activities, but there is so much to be done by one person, and also there is so much administrative work.
Lack of supplies	Primary care personnel: doctor	In primary care clinics sometimes we a feel our hands are tied, because sometimes we do not even have medicines, so to do specific studies is very difficult.
Insufficient infrastructure	Primary health care: social worker	We are very tight as you can see, there are no spaces, obviously the planning of this unit was a result, as in many cases, of an emergency situation, and there is no planning for the future, they do not have that kind of vision, that the population is going to keep growing.
Lack of personnel	Primary care personnel: doctor	I was in a health center and the nurse was suddenly called to cover a different nursing activity, she was called from one day to the next. So, if you have programmed activities with a nurse, you cannot perform them as planned because suddenly they have sent him/her to another site.
Lack of interest	Primary health care: social worker	I am sincere, if someone is not interested, it will not work. For example: there is a partner who proposes a new program, but if the health care personnel is not interested, how are they going to collaborate with the program?
Distant medical appointments	Male lay participant	And then, you have available appointment slots for consultations at the hospital in more than a month’s time. Now, for example, there are no slots available until March of next year, there are no available slots since October.
Long waiting time	Male lay participant	It takes a whole day at the health center to get an appointment, one has to spend all day there.
Administrative procedures	Primary care personnel: doctor	Lately I don’t go to my social security clinic anymore because there is too many people, they cannot see us all. One arrives, takes a turn, and they send you to stand in line to wait to see a doctor, but not your family doctor. If you bring studies you do not see your doctor, you have to see another one, it’s such a big mess! So, what is the point of going there?
***Interpersonal level***		
Peers’ fear of colonoscopy	Primary health care doctor	Well, it’s fear, right? Fear of the procedure. More if a neighbor or relative tells them that colonoscopy is very painful. I think that would be a barrier.
***Individual level***		
Fear of colonoscopy	Female lay participant	But that study is dangerous, right? You can die there or something? ... because they put a tube all the way up to here... I’m afraid I could die.
Fear of finding out about a serious disease	Female lay participant	I believe that when people already have certain symptoms and they need to have the study done, they have fear to be told they have a disease. That is what happened to my son-in-law. He was afraid to be told that he had cancer... that they could find he had something bad.
Embarrassment of having a colonoscopy	Female lay participant	I would be embarrassed that a doctor sees me, that a doctor introduces a camera, through my rectum.
Lack of time for use of health services	Male lay participant	Those comments are very frequent among my friends ... “They fired me from work, I went to the doctor, and it turns out that they fired me and right now I am unemployed.” So I think: I have a job and I will take care of it. So that’s what worries me, you can do the study, but how are you going to detect an illness in your body if you can’t skip work or you can lose it? For example, now I have about four or five years since in this job which was hard to find.
Procrastination	Female lay participant	I have invited people to many programs and they tell me, oh yes, let’s see, some day, once I decide then I’ll go, let me see when I have time.
Disinterest towards health	Primary health care: social worker	I also believe, sorry to be very rough, but I think that some patients have no strength. You call them for follow-up and referral and: “I haven’t been able to go, I am not interested, the hospital is too far away”. I tell them, it’s your health, I mean cancer is a priority, but people do not come, even if they have dysplasia or BIRADs 4, they don’t come. They make up a thousand excuses.
Preference for traditional medicine	Primary health care: social worker	Many times there are people who have already arrived here from the countryside and they are not used to the use of medicines, they want to return to natural treatments, naturists, herbs, teas. There are diabetic who say “I am going with a naturist doctor and he tells me that he will cure me”… They swear that they are going to be cured of diabetes and when they come back to see us they come in very uncontrolled states.
Distrust in public medical services	Male lay participant	Why go to IMSS (main public institution available for the formally insured)? If they don’t give an adequate answer to one’s illness, then why see them? It’s better this way. I prefer to look for a doctor close-by. Even if I have to pay, it is better quality and it doesn’t take all day long to get an appointment
Reluctance to use health services	Male lay participant	Because one as a man has a little bit of pain and we think that it will disappear, and we continue working, but you don’t know if there is a disease that is advancing, it is progressing. It is until it is well advanced that you regret you didn’t seek care earlier.
Low perceived risk of CRC	Male lay participant	Well, one as a man does not know, because the ladies get breast cancer. I’ve heard only women with cancer, not men.
Non-acceptance of biopsy	Endoscopy medical personnel	There were 8 patients who still did not come to schedule their colonoscopy study, so I started to communicate with them and one of them told me that she already knew about the program, but she did not want a colonoscopy because she doesn’t want a biopsy.
Lack of self-care	Primary health care: social worker	Thirty percent do not return for their results, they do not do it because of indifference, lack of self-care, they are not responsible of their own health.
Low self-esteem	Female lay participant	We carry so much as women, so much stress. Illnesses are for us so if we, as women, do not love ourselves, then we’ll die tomorrow.

**Table 4:** Participant quotes of perceived facilitators to colorectal cancer screening

Code	Informant	Quote
***Health policy level***		
Appropriate communication campaigns	Endoscopy unit medical personnel	I think that if you educate people, and the program is promoted through radio, television, newspapers, if the information is appropriate and concrete, talking about a general population, this could help get more people to accept the procedure.
***Social context level***		
Participative population	Primary health care personnel: social worker	I believe that this population is participative, if you ask for their support, and offer a service, they organize themselves and they participate.
***Organizational level (health services)***		
Doctor communication skills	Primary health care personnel: doctor	Well, there are certain skills a doctor should have: empathy, the power of communication so that there is no barrier with the patient, the skill to talk with the patient and to be persuasive.
Good experiences of health service use	Female lay participant	I have been very lucky because I have always been treated very well in social security, there my daughters were born, my husband has had several surgeries, in fact, I have run with luck that my family doctor identified myself
Good working environment	Primary health care personnel: nurse	I have felt very good here, the work environment is very good, all my colleagues focus on their work and everyone is in charge of doing their part, the atmosphere is very good
CRC screening promotion at clinics	Primary health care personnel: nurse	The promotion could be made in the health center, there could be an exclusive module for this, so that patients are given exclusive attention.
Good doctor-patient relationship	Female lay participant	I have been very lucky with the attention I have received at IMSS. I delivered my daughters there and my husband has had several surgeries, and they treated us very well, in fact, I have been lucky. My family doctor knows who I am, even though they treat many patients and usually they don’t know the patients’ names, my doctor recognizes me and we have a good doctor-patient relationship.
Motivated health care personnel	Primary health care personnel: doctor	Having that kind of patients motivates us to keep working, reading, studying and everything, because many things or diseases that we, or I personally did not think where so common, but actually are, I think it is important to go back and study them, that motivates me.
***Interpersonal level***		
Social Support	Female lay participant	And the family sometimes encourage you, family support is important to encourage you
Knowing someone who had cancer	Male lay participant	I have a testimony from a friend who died 1 year ago. He was feeling ill, they say the body gives warnings, but sometimes that is not so, or we don’t give these warnings importance. With my friend, I don’t know how long he felt ill for, but by the time he sought medical care, they found he had advanced stomach cancer.
***Individual level***		
CRC information	Male lay participant	Well, I think this is very important, just like right now with the information and attention we are getting from you. I think that people would be motivated if they hear this information and think it is important that they get the test to know if they have something, right?
Interest towards health	Primary health care personnel: social worker	I believe that the interest in their health is very important, the interest in their health would be a patient’s strength.
Perception of FIT as easy to do	Female lay participant	The test is not difficult to do, I can do it by myself and nobody will know, nobody will notice. I take my test where I have to and done.
Information on FIT test benefits	Female lay participant	It is a very, very effective and necessary test. If people don’t know that there is such a test, they cannot prevent cancer.
Perceived risk of CRC	Female lay participant	Anyone can get this illness, anyone can get sick, right? I do not know the reasons for this, sometimes there is no reason and people get cancer, so if we have the opportunity to get information about the disease, well, it is good to know what is happening.
Previous experiences with serious diseases	Male lay participant	After what I already suffered with a disease that I had in the past, the truth is that I would do the colonoscopy.

### Perceived barriers to CRC screening

#### Health policy barriers

Barriers at this level were identified only by healthcare providers employed at the Endoscopy Unit at INCan, who reported numerous barriers to the expansion and sustainability of INCan’s current CRC screening program. One of the main barriers mentioned was the lack of interest from decision makers. “This kind of program could fail due to the lack of support of decision makers. I see that many authorities are not interested in colorectal cancer. They don’t think for a minute about the possibility of having a prevention program. Some authorities in our hospital are aware of the relevance of this, but not all…” (Endoscopist). Other barriers included budget constraints, insufficient promotion of CRC screening, and dissemination of inaccurate information about CRC in mass media campaigns.

#### Social context barriers

Poverty was the most commonly perceived barrier to uptake of CRC screening, as reported both by community participants who would be the targets of screening and the primary healthcare providers who serve this population. Participants in all groups consistently brought up concerns about costs of tests and described living conditions that prevail in the area and the daily difficulties that patients face to cover basic needs (e.g., drinking water, food, and medicines). Among elderly male participants, most complained about the challenges of finding work at an advanced age. Among the female participants, several reported being completely dependent on government programs for food and medical care.

Belief systems about cancer, health in general, and medical treatments were identified as another social context barrier. For example, community participants spoke about the commonly shared fatalistic view of cancer as a “death sentence” accompanied by suffering, pain, and expensive treatments that have a negative economic impact on the family. They also spoke about a common attitude of carelessness towards one’s health, reflecting the perception that many take health for granted. They shared the observation that many do not prioritize preventive healthcare and postpone health service utilization until symptoms are severe. Moreover, the role of gender with regard to beliefs about health was consistently mentioned by participants from all groups, with the shared impression that men are less likely to utilize healthcare services than women. Many attributed this to men being less concerned about health than women. Additionally, community participants thought that having a colonoscopy would be harder for men to accept due to the anal penetration associated with the procedure, with possible sexual associations. In the words of one of our male community participants: “For men my age and older, it is very difficult that they will agree (to having a colonoscopy), because they are going to say that they are being raped. They will say: at this age they are going to rape me with the finger? No, you are crazy, I tell you the truth….”

One more barrier related to gender beliefs that could potentially affect the uptake of colonoscopy by women in Mexico is machismo or a sense of masculine pride that includes control over the female partner. Some participants described the possibility that some men may forbid their wives from seeking medical care, particularly if the doctor is a male and the consultation could require a woman to show intimate parts of her body. “We are far from many things, because first we start under the assumption that us women are destined to be nothing more than a housewife, and if you have a controlling and jealous husband, forget it, how do you think you are going to go get this test done?” (Female community participant).

Numerous participants in all groups perceived the lack of knowledge about CRC and CRC screening among community and primary healthcare participants as a relevant barrier. In particular, community participants lacked even basic knowledge about CRC and saw lack of knowledge as a barrier to participation in screening. Few community participants had heard of colonoscopy and knowledge of the procedure was limited. None of our community participants had heard about FIT as an option for CRC screening. The primary healthcare personnel possessed little knowledge about CRC and options for screening.

Finally, there were characteristics of the community members that primary healthcare providers perceived as barriers for a successful implementation of a CRC screening program. The health workers perceived the population they serve as poorly educated. They described it as challenging for community members to understand instructions for participation in diagnostic tests, management, and follow-up of chronic conditions (e.g., diabetes). “The patients have low levels of school education, people with maximum 3 years of primary school, so we face many complications because they do not understand how to take the treatment or how to take samples for lab tests and therefore for adhering to treatment and follow-up…(Primary care doctor). Also, the primary healthcare providers perceived the community as accustomed to participating in health programs in response to incentives (e.g., food parcels), which is a common practice with the delivery of social programs in Mexico. The primary care participants also described street violence as a barrier to providing outreach in certain neighborhoods. They also commented on the community’s cultural diversity, with migrants from different ethnic origins, which in their view further complicates the primary care personnel’s usual outreach activities. Finally, primary care providers reported high community turnover due to migration from and to other states in Mexico or even change of residence within the city as a factor which could pose challenges to successful follow-up of individuals with positive FIT results.

#### Health service organization barriers

Community participants perceived the following potential barriers to participation in CRC screening: (1) previous experiences of patient abuse or mistreatment in healthcare; (2) poor quality of health services; and (3) challenges in doctor-patient communication. Several participants, including primary care physicians, shared negative personal experiences as patients in public health services that have subsequently prevented them from seeking care. These included perceived poor quality of care as well as stories of patient abuse where participants felt they were discriminated against due to their low-income status or appearance. “It is true that security guards (at hospitals) are sometimes very bad, completely inhuman, right? They say: you are not from around here, you need to show me your health service identity card, if not then look elsewhere... If it is already a hardship to get to one hospital, then imagine having to move from one place to another?...” (Male community participant).

Finally, community participants complained about not getting satisfactory explanations from healthcare providers about their health conditions, details for the rationale of medical recommendations related to screening and treatment, and wording that is easy to understand. Also, they said they wished doctors were more empathetic towards their life experiences.

At the primary care clinic level, the most prominent barriers perceived by our two groups of health care personnel participants (primary care and endoscopy unit) were as follows: (1) lack of CRC knowledge among the primary care providers; (2) work overload in the primary care clinic; (3) insufficient infrastructure, personnel, and supplies; and (4) resistance to or lack of interest among primary care personnel in participating in new programs. The second barrier listed appeared to be a central issue: a majority of healthcare providers identified work overload as a significant problem, articulating that it would be very difficult to recommend screening during patient visits due to numerous competing medical priorities, short consultation times during patient visits, and a high administrative workload. ”It’s only one nurse, one doctor, one social worker and a lot of people, so obviously you cannot cope with the attention for all the patients. You have to organize your times, because there are so many activities. If a procedure gets a bit complicated or takes you a little extra time, you will not be able to perform two or three pap smears. I would like to be able to organize my activities, but there is so much to be done by one person, and also there is so much administrative work...” (Primary care doctor). Additionally, healthcare personnel referred to the daily challenges of doing their job in the midst of insufficient infrastructure, lack of supplies, and inadequate staff. Also, they perceived the lack of interest among staff and their resistance towards participation in new programs as an expression of fear regarding impact on an already heavy workload.

Finally, community participants described as potential barriers (apparently based on previous experiences) the long waiting times for referrals to other hospitals. “And then, you have available appointment slots for consultations at the hospital in more than a month’s time. Now, for example, there are no slots available until March of next year, there are no available slots since October.” (Male lay participant). Additionally, they mentioned complicated administrative procedures and long distances for transportation to the health services could be barriers for screening completion. Although INCan is located only 10 km away from the community, distance was perceived by the community population as a barrier specific to getting a colonoscopy at the Endoscopy Unit of INCan, as public transportation is limited and can take much longer than private transportation.

#### Interpersonal barriers

At the interpersonal level, one of the endoscopists mentioned that negative colonoscopy experiences among peers might influence the uptake of this procedure. “Well, it’s fear, right? Fear of the procedure. More if a neighbor or relative tells them that colonoscopy is very painful. I think that would be a barrier…” (Endoscopist). Among our community participants, nobody knew anyone who had previously undergone a colonoscopy; however, one female participant narrated to the rest of the group a horrible experience with the sedation of her son during an endoscopic procedure and expressed her fear of submitting herself to something similar.

#### Individual barriers

One of the most evident barriers was lack of awareness about CRC among community participants. A majority of participants openly acknowledged not knowing anything about CRC and were unable to identify the location of the colon. Once information on CRC, FIT-based screening, and colonoscopy was provided, the most commonly reported barrier was fear. Participants discussed the fear of finding out they have a serious disease like cancer. Three additional kinds of fear came up in relation with colonoscopy: (a) fear of pain; (b) fear of not knowing what to expect during the procedure, even dying because of it; and (c) fear of embarrassment regarding the actual colonoscopy procedure, particularly among the male participants. Some of our participants perceived it as a dangerous procedure: “That study is dangerous, right? You can die there or something? ... Because they put a tube all the way up to here... I’m afraid I could die…” (Female lay participant).

Community participants also reported lack of time for utilizing health services due to personal obligations and daily life activities. Male participants mentioned fear of losing their jobs, and female caretakers consistently put their families’ needs before their own. Respondents explained that community members have too many competing responsibilities, and preventive health care is not a priority.

According to participants, preferences for traditional rather than allopathic medicine, particularly among people who migrated from rural areas to Mexico City, were identified as a potential barrier to participation in CRC screening. Reluctance to use health services due to distrust of healthcare providers was consistently reported. In the voice of one of our community participants: “Why go to IMSS (main public institution available for the formally insured)? If they don’t give an adequate answer to one’s illness, then why see them? It’s better this way. I prefer to look for a doctor close-by. Even if I have to pay, it is better quality and it doesn’t take all day long to get an appointment.” Other barriers that were mentioned were lack of self-care, low self-esteem, procrastination, disinterest in health, and low perceived risk of CRC.

### Perceived facilitators for participation in CRC screening

#### Social context facilitators

Health workers at the primary care clinic perceive that the population they serve has been highly engaged in other health programs offered in the past. They perceive that this openness of the community to participate in health programs could facilitate uptake of CRC screening.

#### Health service organization facilitators

Facilitators perceived by our community participants at this level were having good doctor-patient relationships, having satisfactory communication skills among doctors, and having history of positive experiences with health service utilization. Primary care personnel commented on the need for appropriate work environments. A majority reported that motivation of the primary care personnel to participate in the CRC screening program was key to successful implementation of the program.

#### Interpersonal facilitators

Some community participants reported that knowing someone affected by cancer, particularly a family member or a close friend, would be a motivation to participate in cancer screening. Social support was also considered an important facilitator. Many reported that it would be easier for them to participate in screening if a family member or friend encouraged them to do so or shared with them a personal positive experience. “The family sometimes encourages you. Family support is important to encourage you” (Female community participant).

#### Individual facilitators

Almost all participants expressed that access to information on CRC and the benefits of screening is an important facilitator. The community participants were very interested in receiving more information about CRC screening and prevention. The information they received in the focus groups made them feel at risk for CRC (risk perception) and in control of detecting it early (perceived benefit of screening test); several mentioned this information as a motivation to participate in CRC screening. Other potential facilitators were that the participants perceived sample collection for the FIT test and return of the kit to the health center as simple procedures. Knowing that the test could be done at the privacy of their homes was seen as an advantage. “The test is not difficult. I can do it by myself and nobody will know, nobody will notice. I take my test where I have to and done” (Female community participant). Finally, having personal experiences with serious illnesses came up as a facilitator. Some participants reflected upon their own negative health experiences and said that they were willing to participate in any screening activity that would prevent them from additional suffering due to health issues.

### Perceived useful implementation strategies to promote CRC screening

Participants also mentioned several implementation strategies that could enhance CRC screening uptake. All types of participants recurrently mentioned that for CRC screening participation to be successful, FIT tests and colonoscopies should be offered at no cost. Additional suggestions for implementation strategies highlighted the importance of involving community-based clinics, including (1) promotion of CRC screening at local community clinics, (2) recommendation of CRC screening to all patients older than 50 years by primary care physicians, (3) availability of the FIT kits at the local clinic, and (4) ability to receive completed FIT samples at the local clinic. For uptake of colonoscopy, several community participants suggested the procedure be done by a physician of the same gender. Health workers at the Endoscopy Unit suggested mass media campaigns to inform the general population about the benefits of CRC screening and who should be screened. In order to improve their CRC screening knowledge and communication skills, primary care personnel suggested the use of short informative videos. Also, they commented on the importance of observing others to learn medical procedures, which could also be applied to learning to more effective communication skills to explain and promote CRC screening.

## Discussion

Our study identified multiple barriers and facilitators to successful implementation of a FIT-based CRC screening program in a low-income urban community in Mexico City. The main barriers at the social context level were poverty, health literacy, and community health and gender-related beliefs. At the health services organization level, the lack of knowledge of CRC among health care personnel and common perception of poor quality of health care services provided at public facilities were identified as major barriers. We identified lack of awareness about CRC risk and fear of serious disease as the preeminent barriers at the individual level. The major perceived facilitators for a CRC screening program were health education on CRC screening and access to screening tests at no cost to the patient.

Previous studies have reported similar barriers to the ones observed in the current study. At the social context level, health beliefs and attitudes, like fatalism [23, 24, 38], sexism, and stigma related to the digital rectal exam [39], have been reported in several studies. At the level of the health system, the following barriers have been previously reported: negative experiences with healthcare services or poor perception of the quality of healthcare provided by personnel [40, 41], insufficient explanations by doctors about the evidence to support use of the screening studies [42], lack of confidence in the health system [26, 40, 43], difficulties with appointments, referrals, long waiting times, and failures in reminders [44, 45], and access problems due to health insurance and test costs [41]. At the interpersonal level, lack of social support has also been reported as a barrier to CRC screening participation [46]. Finally, at the individual level, previously reported barriers include lack of knowledge about detection and disease [40], underestimation of CRC risk [47, 48], procrastination [29], fear of a cancer diagnosis [25, 29, 39, 44, 49, 50], fear of discomfort or pain during colonoscopy [25, 27, 51], and shame about getting a colonoscopy [25, 27, 51]. Our study participants perceived the removal of financial barriers and implementation of educational interventions for patients and providers as hypothetical facilitators. Both of these have also been found to be among the most successful facilitators of CRC screening in other countries where screening programs have been piloted or are already in place [26, 29, 30].

Awareness of CRC screening was very low among our participants at baseline, but once they were given information on CRC, the benefits of screening, and the details regarding the screening tests, interest in participating in the collection of stool samples for the FIT was high, and no concerns regarding the actual procedural aspects were expressed. Contrary to research findings from countries such as Spain [25], the Netherlands [28], the UK [52], and the USA [29], our study subjects did not report taboos or unpleasantness of handling stool samples as a significant barrier to patient participation.

Qualitative research studies conducted prior to program design and implementation, engaging stakeholders at multiple levels of the SEM, and aimed at identifying local barriers and facilitators, can provide valuable information to increase the likelihood of successful program adoption, implementation, and sustainability. Our findings highlight the need for culturally appropriate CRC screening interventions that address perceived barriers and facilitators for successful implementation. First, it is relevant to consider the characteristics of the target population. Individuals and populations afflicted by poverty are likely to prioritize fulfillment of basic needs over preventive services [53, 54]. Once participants in our study received information regarding CRC screening, most expressed a willingness to participate in CRC screening, although test costs were perceived as a very important barrier. Therefore, access to tests free of charge needs to be guaranteed if people living in limited-resource settings are to be targeted by screening programs. In the Mexican context, diagnostic colonoscopies are not currently covered by the national health insurance plan, and efforts are ongoing to address the critical need for downstream capacity and coverage for the diagnostic colonoscopies that are necessitated by a positive FIT result.

Second, the knowledge gaps about CRC risk among members of the primary healthcare team, including physicians, emerged as a very important barrier to target prior to implementation of a CRC screening program. Primary care personnel need to be educated about the relevance of CRC: the epidemiologic burden, the role of screening for prevention and early detection and the specific feasible screening recommendations, as well as cultural competence and communication skills relating to promotion of screening [55]. Finally, increasing awareness of CRC among the lay population will be critical to creating familiarity with a recommendation for screening [56]. Addressing these barriers thoughtfully and sequentially will be necessary to ensure that access to screening and diagnostic tests are well-established before promoting awareness among the at-risk population, in order to avoid escalation of a health need that the health system is not prepared to meet.

Some limitations of this study need to be acknowledged. First, study participants were instructed by focus group facilitators to speak on behalf of cultural views that would be representative of their communities, though participants were not restricted in the actual discussions and may also have provided personal views. However, we believe this information is valuable as well, as personal views are often a reflection of shared cultural values. Also, we recognize that there is important demographic and socioeconomic heterogeneity within Mexico City that may not be reflected in our sample. The neighborhoods sampled were among the poorest in Mexico City, and results may not be entirely generalizable to communities with higher income levels. However, to address this issue, we purposefully sampled individuals of different gender and age from different neighborhoods surrounding the clinic. We also included a multidisciplinary sample of healthcare personnel who would be directly involved in implementation of a community-based CRC screening program. This purposefully sought heterogeneity of our 50 participants allowed us to achieve data saturation. Because poverty remains highly prevalent in Mexico City, the types of barriers and facilitators perceived by our informants are likely to be representative of a large proportion of neighborhoods across Mexico City.

## Conclusions

We identified three main barriers to CRC screening in a low-income, urban community in Mexico City: (1) a need for free provision of FIT tests and diagnostic colonoscopies, (2) training for primary health care personnel, and (3) promotion of CRC screening awareness among the target population. As we consider steps necessary for the implementation of a successful CRC screening program among marginalized communities in Mexico City, we aim to create an intervention that is implemented through a well-coordinated multidisciplinary team that includes all these complementary elements. Our future research activities will aim to address each of these three barriers in a stepwise fashion through a multi-level approach that engages policy makers, stakeholders within multiple healthcare settings, and community leaders and members.

## Data Availability

Data available on request from the authors.

## Abbreviations

CRC: Colorectal cancer
FIT: Fecal immunochemical test
gFOBT: Guaiac-based fecal occult blood test
HBM: Health belief model
HIC: High-income countries
INCan: Instituto Nacional de Cancerología (Mexican National Cancer Institute)
LMIC: Low- and middle-income countries
PRECEDE-PROCEED model: Predisposing, Reinforcing and Enabling Constructs in Educational Diagnosis and Evaluation - Policy, Regulatory, and Organizational Constructs in Educational and Environmental Development Model
SEM: Social ecological model
